# New Working Capabilities for Coping With COVID Time Challenges

**DOI:** 10.3389/fpsyg.2022.814348

**Published:** 2022-04-21

**Authors:** Ezio Fregnan, Giuseppe Scaratti, Leonardo Ciocca, Silvia Ivaldi

**Affiliations:** ^1^Faculty of Economics, Catholic University of the Sacred Heart, Milan, Italy; ^2^Department of Human and Social Sciences, University of Bergamo, Bergamo, Italy; ^3^Department of Psychology, Catholic University of the Sacred Heart, Milan, Italy

**Keywords:** digital transformation, pandemic crisis, work and organizational culture, managerial competences, actionable skills, agile work

## Abstract

The COVID-19 pandemic played as a booster to the cultural, social, and economic transformations triggered by the 4.0 Industrial Revolution, increasing the diffusion and employment of technological devices and requiring to reconsider the traditional approach to work and organization. Dealing with an emblematic organizational case, the article highlights the main key capabilities requested to face the current scenario, suggesting transformed attitudes needed to cope with the unfolding complex, uncertain, changing digital and blended world. The findings, gathered through an extensive survey involving 500 people who started working at a distance during the 2020 lockdown period, underline the main actionable skills to be achieved for enhancing agile work, hybrid professional roles and new work, and organizational and managerial cultures.

## Introduction

During the past centuries, the world we live in has faced several phases that significantly impacted every aspect of human life: culture, society, and economy. There has always been a strong connection between human beings and technology, as starting from the First Industrial Revolution progress and innovation have played a crucial role in originating new conceptions of society, from rural/feudal to industrial/capitalist and then to industrial/tertiary. The key drivers at the core of such transformations have always been automation and connectivity, allowing to increase the productivity at the global level (Blinder, 2008; Prisecaru, 2016; Schwab, 2016; Corazza, 2017; Park, 2017; Caravella and Menghini, 2018). Furthermore, the last decades have experienced a new set of changes, mainly originated by the digital revolution, that has introduced artificial intelligence, big data, robotics, and Internet of Things as its peculiar elements (Schwab, 2016; Caruso, 2017), with repercussions on the physical (e.g., autonomous vehicles, 3D printers, advanced robotics), digital (e.g., IoT, platforms, IoS), and biological spheres (e.g., artificial intelligence for genetics, biology, and related applications). This new phase is increasingly transforming our lives and has been recognized as the Fourth Industrial Revolution since when the World Economic Forum (WEF) focused its attention on it in 2016 (World Economic Forum [WEF], 2016). We are not fully aware of its potential and how to deal with it yet, but many debates are discussing the current situation, highlighting both the advantages and risks it implies.

A first remarkable point is that there is no general agreement on this definition to describe the current scenario. According to several authors, we are, in fact, just experiencing the effects of the Third Industrial Revolution, and its evolution is imminent (Blinerd, 2006; Rifkin, 2016). Schwab identified three criteria at the basis of a future revolution we are going to live (Schwab, 2016): speed (thanks to technologies in continuous development - our heterogeneous world is vastly interconnected), range and intensity (each dimension of our life constantly changes due to innovative combinations of different technologies), and impact on the systems (such a radical transformation can impact on systems at many levels, transforming even countries and the global society itself). Moreover, there is some controversy surrounding the long-term effects generated by the Fourth Industrial Revolution. Optimist authors came to the conclusion that the damages caused by new technologies will be then reabsorbed in the long term, thanks to the increased opportunities originated by digital innovations. Their analysis has not found general acceptance from the pessimist authors, who predict an unstoppable fall that will lead us to the end of work and, consequently, to ever larger inequalities at both global and intra-national levels (MacCarthy, 2014).

Undoubtedly, the world of work requires a new approach, and Industry 4.0 is characterized by a renewed conception of the manufacturing processes, decentralized and adopting systems based on information and communications technologies (TIC) (Park, 2017). The inevitable purpose of organizations in such a challenging context is both to survive and grow in the long term, remaining competitive and innovative, adapting to a number of situations never experienced before to a similar extent.

An additional element introduced by the literature (Kovacova and Lãzãroiu, 2021; Nemţeanu and Dabija, 2021; Riley et al., 2021; Wade and Vochozka, 2021) is the relationship between sustainable Industry 4.0 wireless networks and big data-driven decision-making processes in sustainable organizational performance. In fact, it has been noted that Internet of Things and artificial intelligence data-driven systems are able to support a sustainable Industry 4.0 wireless network and a digitized mass production in cyber-physical smart manufacturing (Wade and Vochozka, 2021). Specifically, a wireless communication system supports deployment through real-time supervision of production resources and tools (Kouhini et al., 2021). This data-driven monitoring and the predictive analysis ensure product traceability, production maintenance, and performance improvement (Kovacova and Lãzãroiu, 2021). Deep learning processes arise and lay the ground for cyber-physical production networks and intelligent planning in a sustainable Industry 4.0 (Kovacova and Lãzãroiu, 2021; Riley et al., 2021).

Employees are constantly being demanded to be more and more flexible, fast, and ready to deal with the effects of technological progress, impacting the complexity and dynamicity of their working activity (Carroll and Conboy, 2020). The traditional approach to work has to be reconsidered; new competences and tools are needed to deal with “digital employees,” “digital management of workforce” and “digital work” (Strohmeier and Parry, 2014).

While trying to cope with such a challenging scenario, in 2020, we had to face a sudden and unexpected event causing a global crisis that is still destabilizing our world at economic, social, and cultural levels. Besides all its dramatic consequences, the COVID-19 pandemic is also heavily accelerating the diffusion and employment of technological devices (smart work, apps, analytics, etc.) to respond in a time of emergency (Davison, 2020; Philips, 2020). Aiming to find a new balance in every aspect of the current surrounding context, characterized by bewilderment, confusion, and concern, it is essential to explore some key issues at stake in such a renewed work environment.

The paper refers to the new capabilities that the pandemic has revealed as necessary to deal with a profoundly changed work environment in a very short time frame in order to investigate if their potential can contribute to the transformed attitude needed to face the present scenario, as well as in the future when the pandemic can be considered overcome. The article aims to answer the following research questions:

• *What key capabilities have emerged as necessary as the result of a sudden and radical transformation as the COVID-19 pandemic outbreak?*
• *Can these capabilities represent the key elements of a new culture of work for the future, even after the pandemic?*

The first section of this study proposes an analysis of the theoretical framework related to the new culture of work and the capabilities identified as its distinguishing elements, describing the need to implement new approaches, to be more flexible, and to find innovative solutions in order to adapt to an unexpectedly transformed scenario. The actions undertaken to collect empirical evidence are then described, focusing both of an external desk-based analysis about the impact of the COVID-19 pandemic and a survey involving 500 workers who experienced remote working during the 2020 lockdown period. The conclusions are drawn in the final section, where some considerations, comments, limitations, and hints for future research are provided.

## New Work Culture and Capabilities: Theoretical Framework

Taking into consideration its effects at different levels and noticing how interconnected and automated the industrial production has become, thanks to digital technologies, it is understandable why the current scenario has been identified as a new phase of industrial revolution. The Industry 4.0 belongs to a new system that is dynamic and ever-changing, no longer based on a linear logic of cause/effect, rather on the connection of technology, immaterial features, and human beings, possible thanks to cyber-physical systems (CPS), combining the physical and digital fields (Chung and Kim, 2016; Park, 2017). The most recent technologies are radically changing our society, creating new needs and perceptions as consumers (Prisecaru, 2016; Daemmrich, 2017; Makridakis, 2017); inevitably, our constant connection is modifying the ways in which we communicate and relate to other people, creating the conditions for a deep identity crisis (Schwab, 2016). It is expected that, in the future, relationships will be increasingly characterized by the hybrid nature of the involved interlocutors (either human or artificial) and environments (either real or simulated), requiring a redefinition of our social attitudes (Falcone et al., 2018).

In a similar scenario, the future of labor can only be perceived as unclear; nevertheless, what is certain is that a new culture of work is needed to face the challenges emerging from the constantly changing globalized reality, where we are all interconnected and influenced from the outside environment (Fregnan et al., 2020). For many decades, organizations have been characterized by a solid structure functioning, thanks to rigorous processes and strict procedures, where each role corresponded to specific tasks and a well-defined position, requiring hard and soft skills, rarely undergoing significant changes. Today, developing a set of established competences is not enough to be an efficient member of a successful organization, as companies are becoming more and more liquid entities, constantly modifying their nature to react to the surrounding solicitations and asking their workforce to act accordingly, acquiring agility and renewed capabilities.

As already illustrated in the introduction, the very nature of the Fourth Industrial Revolution and its consequences in the long term sees opposed positions in the scientific literature; in each case, the attention is constantly focused on the human factor. Undoubtedly, the implementation of automated processes will increase the demand for highly qualified workers able to control new production devices in smart factories, robots will become real assistants for human beings, and machines and persons will represent the workforce of the future, cooperating as never seen before (Bloem et al., 2017). If the technological process is unstoppable, we have on our side the power to raise consciousness of its impact on our daily life (Chung and Kim, 2016; Schwab, 2016; Butera, 2017; Makridakis, 2017). People and organizations have, therefore, to learn how to put together their efforts in a new way, facing a context that is so different and demanding, both aware of the need to learn to work in a different way, as well as to develop a set of new capabilities and attitudes, allowing us to express our potential without suffering the change but being active subjects of the upcoming future.

If in the past the role of each worker required a set of hard and soft skills to be then combined with other roles within an organization, nowadays, job profiles are not enough to identify the competences useful to be active components of the flux in which the company moves. What is missing in this scenario is represented by the capabilities intended as those characteristics, allowing to deal with the current world of work, constantly changing and presenting new challenges at every level. Everyday, working activities are already demanding for a renewed attitude, emerging from the need to act in a smart and agile way, finding innovative solutions and effective procedures to meet the market demand (Garrett, 2013; Chung and Kim, 2016; Schwab, 2016; Morrar et al., 2017; Park, 2017). This phenomenon is also the direct consequence of such a hyper-connected and liquid world (Bauman, 2000), and, for the same reason, it requires an adjustment of the educational system; that should guarantee the possibility to acquire new capabilities in an immersive way by means of meaningful training experiences where the awareness is raised and shared.

The Capabilities in question can be described as a combination of knowledge, ability, and experience to be used by people not just at work but in every aspect of daily life to participate consciously to the era they are living, as well as to build a new future with both intention and full awareness. This is strictly related to the European Reference Framework of Key Competences for Lifelong Learning, required for achieving employment, social inclusion, and active citizenship.

What distinguishes the transfer of this kind of Capabilities relates to a logic of continuous learning (Delors, 1997) and to the active role of learners, cultivating and developing their identity firsthand. At the organizational level, this also means the possibility for the employees to really act on their professional growth, making their voices heard to demonstrate a more self-aware attitude. This appears to clearly indicate that the Capabilities can be considered an integral part of the development of a new culture of work, resulting from the significant transformations taking place in the context of the Industry 4.0. Such a perspective implies interpreting the professional development in global terms, which goes beyond the career path, but impacts the personal development too (Lambert et al., 2012; Zimmermann, 2017).

### The Key Elements of the New Work Culture and Related Capabilities

Seeking to explore how to dwell within the Fourth Industrial Revolution, Ivaldi et al. (2021) highlight the need to link new technologies and sustainability, pointing out the challenge to deal with the spreading digital revolution. Hecklau et al. (2016) underpin four categories of new skills:

● technical (state-of-the-art knowledge, process understanding, media skills, coding skills, IT security);
● methodological (creativity, entrepreneurial thinking, problem-solving, conflict resolution skills, decision-making, analytical skills, research skills, and efficiency orientation);
● social (intercultural skills, language skills, communication skills, networking skills, teamwork skills, ability to compromise and cooperate, knowledge transfer skills, leadership skills);
● personal (flexibility, ambiguity tolerance, motivation to learn, ability to work under pressure, a sustainable mindset, and compliance).

At stake is the need to cope with the transformation of work as an impact of 4.0 Industrial Revolution, accelerated by the pandemic scenario, going beyond the traditional distinction between hard and soft skills, achieving concrete and actionable skills as, peculiarly, human abilities, which represent the great qualitative difference between man and machines, enhancing sustainable hybrid professional and managerial cultures (Fregnan et al., 2020) and ad-hocratic and agile organizational systems (Mintzberg, 2009).

An agile approach to work is particularly foreseen, as representing the more suitable one identified to react the high level of chaos, complexity and uncertainty originated by the general context (Sletholt et al., 2011). Such approach requires adaptability (changing and adjusting a project during the different phases of its lifecycle), visibility (to allow the stakeholders to be aware of all the involved aspects and of the level of complexity), generation of value (essential during the whole process, including the concept of value not only from the economic perspective but also from the ethical, sustainable, and eco-friendly ones) and focus on risks (a reconfiguration of purpose, time and cost are required to manage and reduce them; a joint intervention of institutions, industrial and academic world is essential to guide the technological progress toward the community needs).

In order to explore a relevant example of the capabilities required to face the current scenario, deeply transformed by the digital revolution and the pandemic that made clear the need for their application to the workplace, accelerating a process that was still in its infancy, this article refers to a situated organizational case: Comau, an Italian multinational leading company operating in the automotive sector whose activities are strictly connected to the innovative promotion of new capabilities and organizational culture. The Comau experience (Fregnan et al., 2020; Ivaldi et al., 2021) identified a set of seven capabilities as a contextualized interpretation of the theoretical framework (Hecklau et al., 2016) and as a transfer into practice of the new culture of work, essential elements to fill the gap that has emerged since the pandemic outbreak:

● *Deal with humans*: Living in a world so deeply influenced by technology, the human dimension plays an essential role for both persons having to adapt to the surrounding reality and companies having the intention to survive change. The human nature, along with its needs, has to be put at the center.
● *Deal with technology*: As a natural consequence of the digital revolution, today, it is essential to be familiar with the new technologies, taking advantage of the opportunities they offer. It is an unavoidable requirement for workers as well as it is for organizations to implement them in their processes.
● *Agility*: A new culture of work implies open-mindedness and flexibility; it is fundamental to react quickly and think out of the box, in a creative process that often includes the client/consumer itself.
● *Engagement*: Feeling highly involved, properly balancing passion and rationality, being curious and proactive; all of these elements allow to add something personal to each activity or experience, its true driving force.
● *Collaboration*: Complex activities require the cooperation between people that represent the possibility to increase the effectiveness of a performance both qualitatively and quantitatively.
● *Interdisciplinarity*: The new work culture promotes the integration of different kinds of knowledge in order to broaden perspectives and solutions.
● *Innovation*: Fast and unpredictable changes are constantly produced by the new technologies; therefore, it is indispensable to conceive innovation being always open to the introduction of something fresh, to the experimentation of different solutions, and to the combination of data and information. Today, just imagining new ways can lead to the generation of value.

The above capabilities may represent a suitable and actionable expression of the new culture of work under some relevant elements: people and their needs at the center (*Deal with Human*); the confidence with new technologies (*Deal with Technology*); the importance of being open-minded and flexible (*Agility*); the need of a real passion (*Engagement*); the ability to cooperate with other persons (*Collaboration*); the relevance of combining different kinds of knowledge (*Interdisciplinarity*); and the constant search for new possibilities and solutions (*Innovation*).

### Capabilities in the Time of COVID-19

The progressive development of a new culture of work has suddenly accelerated in 2020 due to the COVID-19 pandemic that put a strain on many working environments not always ready to react in such a short time. The unexpected emergency and the consequent lockdown imposed to contain the infection obliged most of the worldwide population to switch the traditional organization of the activities from working “in presence” to working “at a distance,” entailing a relevant effort both at technologic and organizational levels.

Recent publications have highlighted how the social and organizational evolutions, linked to the COVID-19 pandemic, involve new challenges, concerning the relationship between product decision-making information systems (Nica and Stehel, 2021; Novak et al., 2021) and smart factory performance (Hawkins, 2021) in teleworking-related sustainable Industry 4.0 (Nemţeanu et al., 2021). In fact, as Nemţeanu et al. (2021) argue, the need for social distance implied by the COVID-19 pandemic has generated relevant changes in the work sphere (Nemţeanu and Dabija, 2021), expressed in a massive diffusion of telework, which has enhanced the conditions of social distance and isolation of workers. The authors (Nemţeanu et al., 2021) point out that the dimensions of autonomy and reduced interaction, typical of teleworking, have a significant influence on the ability of self-regulation, task and contextual performance, professional isolation, and counterproductive work behaviors. While it has been noted that marketing and internal communication can increase worker satisfaction and improve performance (Nemţeanu and Dabija, 2021), on the other hand, a good management of the dimensions of autonomy and reduction of social interaction, typical of teleworking, can lead to a performance improvement and to a limitation of counterproductive behaviors. In order to guarantee the same efficiency with respect to the usual one, remote working requires above all familiarity with technical devices but also flexibility, proactivity, and adaptability, in a context where you have to communicate with people despite of the physical distance. In relation to these issues, the literature (Hawkins, 2021; Nica and Stehel, 2021; Novak et al., 2021) highlights the role of product decision-making information systems for a sustainable smart factory performance (Hawkins, 2021) in teleworking-related sustainable Industry 4.0 (Nemţeanu et al., 2021). In particular, the most relevant information systems concern Internet of Things, big data decision-making, and analytic algorithms driven by artificial intelligence (Nica and Stehel, 2021; Novak et al., 2021), real-time sensor networks, and cyber-physical manufacturing (Hawkins, 2021).

The recent crisis has, therefore, forced persons to leave their comfort zones, experimenting new approaches to both their working activity and their relationships, contributing to that diffusion of a new culture of work that had already started in the previous years. A deep cultural change is perceived as needed like never before, with a direct impact on the enterprises that are required to invest significantly in the promotion of both organizational and professional learning. Direct experience and practical knowledge will play a crucial role in such a learning process, allowing to introduce, develop, and integrate innovative technologies, as well as to combine both the exploration and the valorization of knowledge (Lanzara, 2006).

Today, workers and professionals are frequently experiencing uncertainties and contradictions during their daily working activity, and, at the same time, they have to look for and create new solutions and possibilities to act. Such context can well represent both a litmus test and a situational organizer not only to encourage the introduction and diffusion of technical devices but also to drive the cultural change at different levels, aiming to face challenging social issues (Engeström, 1987).

This is exactly the focus of the research: Taking into consideration the COVID-19 pandemic and the sudden and radical transformation it has originated on the work context, we sought to investigate if the seven Capabilities emerged as key elements of this peculiar situation can represent an effective answer even once the pandemic can be considered over. The following section illustrates the methodologies adopted to analyze the current emergency scenario, characterized by such significant changes at every level of our life before moving on to the discussion of the key findings and, finally, to the conclusions.

## Research Design, Context, and Methodological Approach

In order to investigate the current scenario, deeply transformed by an unexpected and sudden event such as the COVID-19 emergency, the study illustrated in this article has focused on two main knowledge objectives: a careful analysis of some impacts generated by the pandemic in question and an observation of the perception related to the key elements of the new work culture during this abrupt crisis.

The adopted methodology has been, therefore, implemented at two different levels:

(I) External desk-based research to explore the surrounding context during a peculiar situation and its consequences on the world of work;
(II) A survey involving workers belonging to different areas who have experienced working at a distance during the 2020 lockdown period.

The external desk-based research has been conducted, taking into consideration the interval of time between December 2019 and May 2020, referring to different kinds of sources: books, magazines, newspapers, scientific papers, online news, and informative websites (Eurofound and the International Labour Office, 2017; AstraRicerche and ManagerItalia, 2020; Baldini and Gori, 2020; Banca d’Italia, 2020; Dattoli, 2020; Europea, 2020; Galli and Tucci, 2020; Gavi, 2020; Il Sole 24 Ore, 2020; Kamps and Hoffmann, 2020; L’Eco di Bergamo, 2020; Simon-Kucher Partners Strategy Marketing, 2020; Telelavoro, 2020; Tessa, 2020).

Aiming to a greater understanding of the examined historical period, the research criteria have resulted in the selection of materials and news, dealing with the same events, recurring in several online and offline sources, as well as mentioned among the data provided by official sources. The final purpose has been to outline a reference framework concerning an unforeseen phenomenon that is generating a significant impact on both production and social systems at the global level.

The second moment of research aimed to put to test the seven Capabilities whose development has been accelerated by the pandemic, verifying their validity in the context of a sudden transformation and, possibly, in the future by means of a survey involving people who have personally experienced relevant changes in their own organizational and working environment. The sample has, in fact, included 500 workers who had to start working at a distance in the months of March, April, May and June 2020, from their home or elsewhere, in a teleworking or remote working mode. The survey intended to collect their impressions and to investigate their points of view in order to find out which Capabilities were shown to be more useful and suitable with the objective of being efficient at work despite the de-contextualization resulting from the enforced isolation.

Starting from a focus allowing to outline the profile of the participants in terms of both their age and working activity, the survey moved then toward the evaluation of the remote working experience from different perspectives, aiming to highlight the differences between professional and personal points of view. The seven Capabilities were then at the core of the investigation in order to examine their role in such a peculiar scenario and to identify possible discrepancies between the working context lived in presence or at a distance, as well as analyzing relevant correlations in the answers given by the participants belonging to different generations. The questionnaire also included two scales for the Capability assessment, contextually described so as to obtain information about the order of importance of these in a relative as well as absolute sense, always referring to the two working experiences under investigation. Finally, the survey aimed to collect practical examples related to the application of the seven Capabilities, as well as to identify if further skills have been perceived by the interviewed as important during the remote working experience.

The survey (Table 1) included 17 questions and was spread out through e-mail channels (shared by means of different corporate communication tools) and social networks (Instagram and Facebook), with the goal of achieving a sample being as diverse as possible, to make the results both balanced and objective. The first section (questions 1 and 2) focused on profiling to classify the participant depending on his/her working activity as well as his/her age, associating the person to one of the 4 proposed generations. The following questions (3, 4, and 5) asked to evaluate the appreciation of the working experience at a distance on the whole from both the professional and personal perspectives to examine at the same time the propensity for a future application. The central part of the survey (questions 6–12) dealt with the evaluation of the seven Capabilities identified, exploring how important each of them was perceived by the individual if related to the working activity on site and at a distance. A couple of questions (13 and 14) asked then to express a preference order for the seven presented Capabilities by means of an evaluation scale, referring to the traditional working context in presence first and then remotely, aiming to collect additional information about the relevance order not just in absolute sense but relative too. The final section of the survey (questions 15, 16, and 17) included two open questions and a Likert scale, all related to the practical application of the Capabilities to obtain a deeper insight. The first open question asked to indicate a concrete example of application for one of the seven Capabilities and to motivate why it was felt as important; the Likert scale was related to the perceived relevance degree of the illustrated Capabilities for the future; the last open question investigated the existence of other competences and skills, not included in the shared list of seven Capabilities, that contributed to making the remote working experience more efficient and effective.

**TABLE 1:** Questions of the survey: *remote working experience during the COVID-19 emergency.*

**(1) When were you born?** ° 1995–2009 (Z Generation) ° 1980–1994 (Y Generation) ° 1965–1979 (X Generation) ° 1946–1974 (baby Boomers) **(2) What is your job?** ° Manager ° Employee ° Teacher/Trainer ° Working Student ° Independent Contractor ° Other **(3) How would you evaluate your remote working experience?** ° From a personal point of view ° From a professional point of view Options: Totally Negative\Negative\Quite Negative\Neutral\Quite Positive\Positive\Totally Positive **(4) Evaluate the following statements:** ° “I like working at home, I can balance my job and my personal life successfully” ° “I like working at home, but I’m exhausted as I work more than enough, at the expense of my personal life” ° “I like working at home, but I perform less efficiently than working on site” ° “I don’t like working at home, I feel alone and unmotivated” Options: I Totally Disagree\I Partially Disagree\I Disagree\Neutral\I Agree\I Partially Agree\I Totally Agree **(5) Given the choice, would you continue working remotely in the future?** ° Yes, every day ° Yes, more than two days a week ° Yes, two days a week ° Yes, one day a week ° Yes, just if necessary ° No **(6) Evaluate the importance of the “Deal with Technology” Capability (*being able to use easily and frequently the opportunities offered by new technologies; being familiar with the technological dimension*) in the following situations:** ° During the traditional working activity on site ° Working remotely Options: Totally Useless\Useless\Quite Useless\Neutral\Quite Useful\Useful\Very Useful **(7) Evaluate the importance of the “Deal with Human” Capability (*being empathic, able to put people at the center; constant attention to the people’s needs*) in the following situations:** ° During the traditional working activity on site ° Working remotely Options: Totally Useless\Useless\Quite Useless\Neutral\Quite Useful\Useful\Very Useful **(8) Evaluate the importance of the “Engagement” Capability (*passion and involvement for the performed activity; “adding something of your own,” act with curiosity and proactivity*) in the following situations:** ° During the traditional working activity on site ° Working remotely Options: Totally Useless\Useless\Quite Useless\Neutral\Quite Useful\Useful\Very Useful **(9) Evaluate the importance of the “Collaboration” Capability (*being able to work together with other people, cooperating valorizing diversity*) in the following situations:** ° During the traditional working activity on site ° Working remotely Options: Totally Useless\Useless\Quite Useless\Neutral\Quite Useful\Useful\Very Useful **(10) Evaluate the importance of the “Agility” Capability (*being flexible, reactive, mentally and operationally open, managing non-liner processes in a creative way; being fast in reasoning*) in the following situations:** ° During the traditional working activity on site ° Working remotely Options: Totally Useless\Useless\Quite Useless\Neutral\Quite Useful\Useful\Very Useful **(11) Evaluate the importance of the “Innovation” Capability (*being able to innovate and experiment new solutions; conceiving new possibilities abandoning the traditional way*) in the following situations:** ° During the traditional working activity on site ° Working remotely Options: Totally Useless\Useless\Quite Useless\Neutral\Quite Useful\Useful\Very Useful **(12) Evaluate the importance of the “Interdisciplinarity” Capability (*being able to integrate different kinds of knowledge belonging to diverse disciplinary fields, multidisciplinarity; creating connections between different disciplines*) in the following situations:** ° **During the traditional working activity on site** ° **Working remotely** **Options:** Totally Useless\Useless\Quite Useless\Neutral\Quite Useful\Useful\Very Useful **(13) Referring to the traditional working activity on site, arrange the following Capabilities from 1 (the most important) to 7 (the least important).** ° **Deal with Human** (*being empathic, able to put people at the center; constant attention to the people’s needs*) ° **Engagement** (*passion and involvement for the performed activity; “adding something of your own,” act with curiosity and proactivity*) ° **Interdisciplinarity** (*being able to integrate different kinds of knowledge belonging to diverse disciplinary fields, multidisciplinarity; creating connections between different disciplines*) ° **Agility** (*being flexible, reactive, mentally and operationally open, managing non-liner processes in a creative way; being fast in reasoning*) ° **Innovation** (*being able to innovate and experiment new solutions; conceiving new possibilities abandoning the traditional way*) ° **Deal with Technology** (*being able to use easily and frequently the opportunities offered by new technologies; being familiar with the technological dimension*) ° **Collaboration** (*being able to work together with other people, cooperating valorizing diversity*) **(14) Referring to working at a distance, arrange the following Capabilities from 1 (the most important) to 7 (the least important).** ° **Engagement** (*passion and involvement for the performed activity; “adding something of your own,” act with curiosity and proactivity*) ° **Interdisciplinarity** (*being able to integrate different kinds of knowledge belonging to diverse disciplinary fields, multidisciplinarity; creating connections between different disciplines*) ° **Deal with Technology** (*being able to use easily and frequently the opportunities offered by new technologies; being familiar with the technological dimension*) ° **Deal with Human** (*being empathic, able to put people at the center; constant attention to the people’s needs*) ° **Agility** (*being flexible, reactive, mentally and operationally open, managing non-liner processes in a creative way; being fast in reasoning*) ° **Innovation** (*being able to innovate and experiment new solutions; conceiving new possibilities abandoning the traditional way*) ° **Collaboration** (*being able to work together with other people, cooperating valorizing diversity*) **(15) Taking into consideration the seven identified Capabilities, choose one or more of them that you find relevant and describe a concrete example in which it has been important during your remote working experience.** **(16) In your opinion, how important will this Capabilities be in the future, at the end of this emergency?** Options: Totally Useless\Useless\Quite Useless\Neutral\Quite Useful\Useful\Very Useful **(17) Are there other Capabilities, in addition to the mentioned ones, that you perceived as relevant? If so, what are they?**

Concerning the sample involved in the survey, it included a very heterogeneous population composed by 500 individuals of different ages, belonging to different working environments and coming from various training and geographical backgrounds, covering both Italy and foreign countries.

**FIGURE 1:**
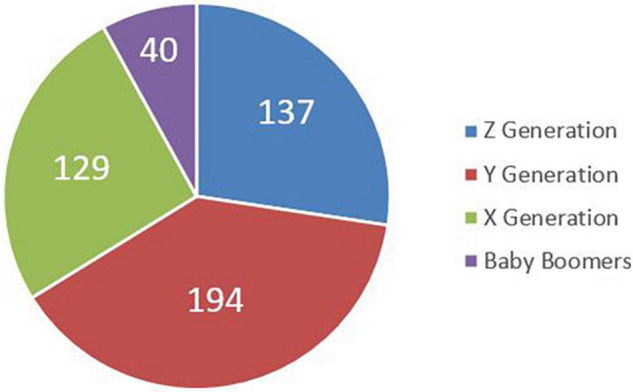
Sample involved in the survey – age.

**FIGURE 2:**
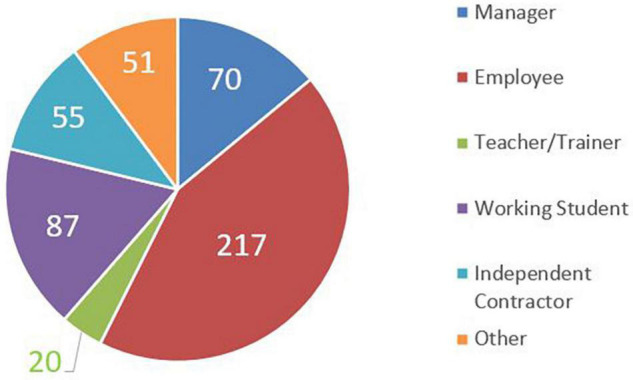
Sample involved in the survey – job.

**FIGURE 3:**
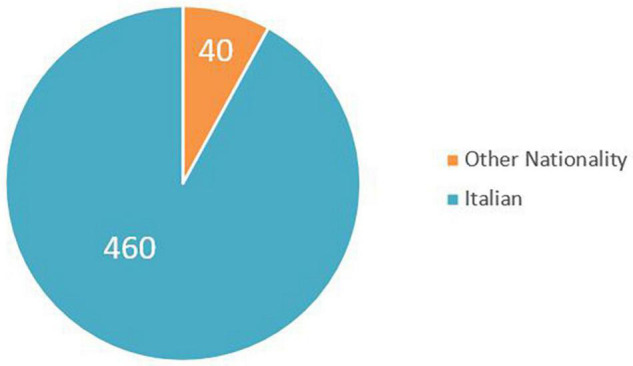
A sample involved in the survey – nationality.

More in detail, the 500 participants in the survey can be classified in Figures 1–3.

The results of the research will be presented and discussed in detail in the following section of the article.

## Key Findings and Discussion

The external desk-based research provided a significant insight into the main phases of the COVID-19 emergency, highlighting its consequences not only at the economic level but drawing particular attention to the deep transformation affecting the way of working worldwide. Such analysis of the impacts of the pandemic helped describe effectively the scenario where workers had to adapt to a new reality, imposing distance above all, creating an unusual context and generating unexpected challenges. A similar situation has proved to be a suitable background for the investigation related to the seven Capabilities of the new work culture, which most of the 500 participants in the survey have recognized as useful elements to be implemented during the current emergency in order to react proactively.

### Findings of the External Desk-Based Research

Aiming to better understand the extent of the COVID-19 pandemic and, in particular, to identify the main impacts of such a phenomenon on the world of work, the external desk-based research has analyzed different sources, combining the data they provided to raise awareness about the context of this study. Both online and offline materials have been examined, including scientific papers, online news, informative websites, magazines, newspapers, and books, focusing on the Italian situation as well as on the global scenario (Eurofound and the International Labour Office, 2017; AstraRicerche and ManagerItalia, 2020; Baldini and Gori, 2020; Banca d’Italia, 2020; Dattoli, 2020; Europea, 2020; Galli and Tucci, 2020; Gavi, 2020; Il Sole 24 Ore, 2020; Kamps and Hoffmann, 2020; L’Eco di Bergamo, 2020; Simon-Kucher Partners Strategy Marketing, 2020; Telelavoro, 2020; Tessa, 2020).

We started to hear talking about the *Coronavirus* in December 2019, referring to an anomalous pneumonia in Wuhan, a city in central China; the contagion extends rapidly and many people die, but the risk is underestimated, and the contamination seems isolated. In the middle of January 2020, a first case is recorded out of the Chinese borders and, by the end of the month, the infection reaches Italy; as the virus can be transmitted from a human being to another one, China starts a lockdown period, imposing the use of surgical masks and prohibiting gatherings of people. Italy declares a state of health emergency; it is the first European country, identifying the COVID-19 as different from the common flu. In February and then March, the situation got worse and worse; the hospitals were close to collapse due to the quick spread of the contagion, and the government decided to “close” the whole country in a lockdown period. By the end of March, the virus reached most European and overseas countries; the United States is the most affected nation, while some emergency initiatives start being adopted to counteract the negative effects at the economic level. Some improvements in the number of infections were first recorded during April and then in May, when Italy entered “Phase 2” and a gradual re-opening allowed the recovery of some activities, always maintaining the safety measures related to the use of surgical masks and the respect of minimum distances.

An analysis of the impacts of the COVID-19 pandemic on the global economy, conducted by the *Simon-Kucher and Partners Strategy and Marketing* global consultancy agency^1^, indicated that just 11% of world economy is flouring, while 58% is in danger. The safer sectors are obviously the pharmaceutical one, as well as those dealing with software, telecommunications, media, and Internet; due to the evident need, the emergency has considerably increased their demand, acting as enablers. The most affected in a negative way have especially been the transport, automotive, and travel sectors, but a significant crisis is also interesting the manufacturing, logistics, advertising, expeditions, constructions and non-essential consumer goods’ industries. Another 17% of enterprises are recording strong positive trends together with difficulties at the operation level. A final result emerging from the same study concerns the remaining 14% of organizations, belonging to the chemical and metallurgic sectors, as well as gas, oil, and energy producers, which are undergoing an overload due to both a decrease or a change in the market demand.

Undoubtedly, the next 2 years will put a strain on the enterprises at the worldwide level, as the expected scenario will be certainly unpredictable and fluctuating; the lockdown period imposed in many countries has seriously compromised the global economy, whose emergency situation has developed in parallel to the health one. Several areas of the world already affected by the food crisis (Middle East, Asia, Latin America, etc.) will be among those to suffer most the consequences of the pandemic, having to manage it among their starving population. All the available examined information also agrees on the fact that the workers involved in the areas that are experiencing greater difficulties are those included in the weaker employment groups, in particular young workers, fixed-term or part-time ones. Such data have also been confirmed by the Bank of Italy^2^, stating that, over the medium term, the COVID-19 emergency will mainly impact the weaker social classes because of the highest number of low-income workers in the most affected sectors, therefore predicting a significant increase in the risk of poverty.

Among the repercussions of this phenomenon, the cultural switch imposed to companies and organizations has accelerated the digital transformation already underway: the emergency management defined new priorities, including health prevention, containment of the anxiety caused by the uncertainties of this crisis, and continuous reorganization. Remote working, e-commerce, e-learning, and robotics have become the only applicable solutions in a context that required sudden evolution, growth, and adjustment to meet the renewed system demand; without warning, millions of workers have started participating in huge smart working and a social collaboration experiment that modified the orientation of our global asset. With people working at home, in many cases, having to take care of their children because of the closure of schools, enterprises had to implement flexible working plans in order to improve their operational processes and facilitate their employees’ life.

It goes without saying that those organizations whose structure was still very traditional and old-school, for instance, several administrations not yet digitalized and mainly using paper documents were unprepared to react timely and consequently suffered the most; at the same time, start-ups and companies that were already implementing the Industry 4.0 attitude, working online from the very beginning, are growing even further or, in the worst cases, did not experience remarkable changes.

If in the past years the digitalization process was considered something probably unavoidable but to be gradually introduced in the world of work, the COVID-19 emergency transformed that slow process of change into an impending need to be put into practice during just few days. Today, we are witnessing an actual revolution of the world of work, which is impossible to prevent at every level, but that, to stay in the game, everybody must accept, allowing technology and innovation to enter our daily activities and change our society, as well as our perception and understanding.

The traditional working mode has to be redefined, taking into consideration that new parameters are, first of all, affecting the whole organizational structure; flexibility and decentralization impose to focus on the effective spread of knowledge and information, a more organic approach is needed to manage complex realities in order to find innovative solutions by means of both the involvement and collaboration of different people (Burns and Stalker, 1961). The management system has, therefore, to move toward the needs of the individuals, who are becoming active participants in the flow, not just performing tasks but also coordinating, understanding, and creating on their own.

This research has focused on working at a distance, including each kind of activity carried out in a place that is not the traditional one, but different naming is used to identify specific situations:

● *Remote working*, or *teleworking*, refers to the possibility of performing the working activity at a distance, maintaining the same working schedule and tasks, using suitable computer tools and portable personal computers connected to the corporate services.
● *Smart working* is instead a sort of evolution of teleworking, indicating a flexible working mode without limitations in terms of time and space; the organization of the activities is, in this case, agreed between an employee and an employer, developed in phases, cycles, and objectives, implemented by means of improved processes and functional technologies.

Working at a distance can obviously have several implications, impacting at the same time on both the individual who performs it and the organization, even reaching the surrounding context with significant impacts on the whole social system.

Advantages for the worker: autonomous management of the daily schedule, reduction of time spent, moving toward the corporate location, more free time and better balance between private and working life, being closer to family and friends, having the possibility to choose the place to live and work.

Advantages for the organization: increased productivity, reduction of costs, more flexibility for the whole enterprise, motivational improvement for some employees.

Advantages for the social system: reduction of traffic and, therefore, of pollution, greater individual productivity and freedom, reduction of costs, and optimization in the space dimension of enterprises.

Disadvantages for the worker: isolation and less direct relationships, risk of workaholism, reduced visibility and consequent less chance to emerge, minor support and guidance received, and loss of separation between the working and the private spheres.

Disadvantages for the organization: redefinition of the corporate structure, difficulty in managing distant workers, increase in the expenses for training and telecommunication, cultural transformation of working processes, and many contracts to deal with.

A first essential condition for an effective implementation of working at a distance is, undoubtedly, the availability of a solid infrastructure network able to meet the current needs; Finland and Sweden are the most digitalized among European countries, while the Italian network is widespread but really slow. Before the COVID-19 pandemic, in 2017, a Eurofund report^3^ had placed our country at the bottom of the list, indicating the percentage of employees who started experiencing teleworking in the EU: Italy, 7%; Germany, 12%; France, 25%; and United Kingdom, 26%. A 20% increase of their number has been recorded for Italy in 2019, which rapidly adjusted its production system in 2020 in order to adapt to the unexpected emergency scenario; such abrupt transformation forced the national economy at every level, imposing the introduction of remote working also in the cases of enterprises not yet planning its application. Italy might not represent one of the most advanced countries of the world, concerning the diffusion of teleworking, but we have witnessed a significant reaction to the sudden transformation originated by the pandemic during the last months, with the adoption of relevant improvements at both technological and organizational levels.

### Findings of the Survey on the Capabilities

The results of the survey involving 500 workers who experienced working at a distance in 2020 have been carefully analyzed in order to investigate how the seven Capabilities implemented as necessary elements of the working scenario during the COVID-19 pandemic are perceived, and if they will be useful also in the future post-pandemic work culture. As the sample included a fairly wide and heterogeneous group of individuals, both from the generational aspect and the kinds of performed activities, such results can be considered an interesting description of the phenomenon, albeit provisional and to be developed; the following paragraphs, therefore, examine in detail the findings emerging from the different sections of the questionnaire.

**FIGURE 4:**
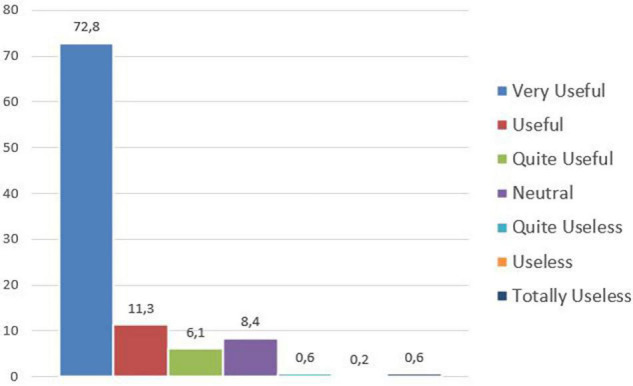
Usefulness of the seven Capabilities in the future.

The question about the usefulness of such Capabilities in the future provided some first relevant results, as an average 90% of the participants answered that those skills will be useful (quite useful/useful/very useful); it is an early provisional support of the research hypothesis, assuming that *Deal with Technology*, *Deal with Human*, *Engagement*, *Agility*, *Collaboration*, *Innovation*, and *Interdisciplinarity* may represent a suitable attitude to act effectively in the post-pandemic working context. The data referring to this question have been collected by means of a Likert scale, composed by seven preference levels, evaluated by the 500 participants as follows in Figure 4.

It is interesting to further analyze the answers focusing on the interviewed generations, which confirm how in all cases the majority of the participants evaluated the Capabilities as useful/quite useful/very useful; just Baby Boomers differ numerically with a slightly lower percentage, which exceeds in any case, 80%.

**Table:** 

	Z Generation	Y Generation	X Generation	Baby Boomers
Very useful	77.3%	70.5%	73%	72.2%
Useful	8%	13%	13.5%	8.3%
Quite useful	5%	7%	4.8%	2.8%
Total	90.3%	90.5%	91.3%	83.3%

The remaining 10% includes 8.6% of the participants who declare neutral and 1.4% distributed among the options useless/quite useless/totally useless, confirming as the only remarkable difference a lower enthusiasm on the part of the older generation of Baby Boomers.

**Table:** 

	Neutral	Quite useless	Useless	Totally useless
Z Generation	8%	0.6%	0%	0.9%
Y Generation	8%	0.6%	0%	0.9%
X Generation	9.3%	0.6%	0.6%	0%
Baby Boomers	9.3%	0.6%	0.6%	0%
Average	8.6%	0.6%	0.2%	0.6%

Taking into consideration the current situation, a dedicated set of questions of the survey asked then the involved sample to evaluate the importance of each specific Capability in absolute sense, first referring to the working activity on site and then at a distance. The same Likert scale above mentioned, composed by seven preference levels, has been used also in this case to investigate the present scenario articulated in the two proposed working modes.

**FIGURE 5:**
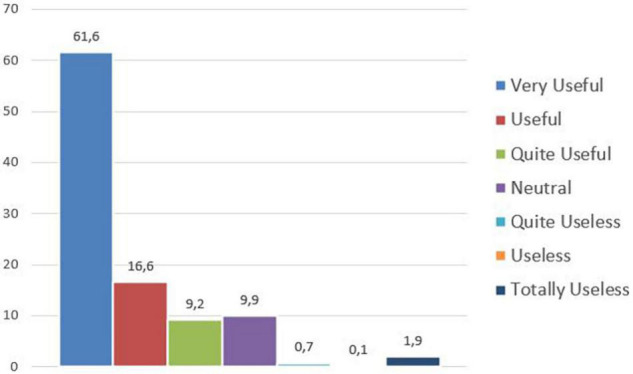
Usefulness of the seven Capabilities during the current situation – working on site.

With regard to the traditional working activity *on site*, an average 87.4% of the participants think that the seven Capabilities will be quite useful/useful/very useful (Figure 5).

**FIGURE 6:**
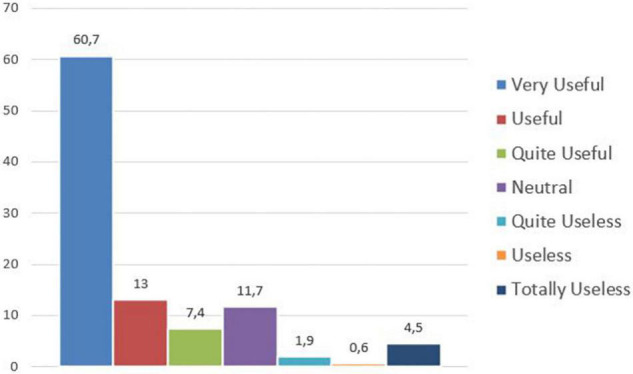
Usefulness of the seven Capabilities during the current situation – working remotely.

Concerning working *at a distance*, an average 81.1% of the participants evaluate their levels of importance as quite useful/useful/very useful (Figure 6).

Without making a distinction between working remotely or on site, an average 84.3% of the sample considers the seven Capabilities as quite useful/useful/very useful.

Further analysis of the collected data referring to the Capabilities has interpreted the seven levels of the Likert scale as points on a range from 1 to 7 in order to investigate how useful each of them is perceived by the workers.

Referring to the traditional working activity *on site*, the following rankings emerge:

**Table:** 

*Collaboration*	6.33
*Deal with human*	6.32
*Agility*	6.28
*Engagement*	6.26
*Interdisciplinarity*	6.14
*Innovation*	6.08
*Deal with technology*	6.01

Taking instead into consideration working *at a distance*, the following rankings emerge:

**Table:** 

*Deal with technology*	6.43
*Agility*	6.15
*Innovation*	6.11
*Engagement*	6.09
*Interdisciplinarity*	5.93
*Collaboration*	5.73
*Deal with humans*	5.47

It is not surprising that the Capabilities considered most useful referring to the traditional working activity on site are the ones concerning human relationships and the human factor, highlighting the increasing importance attributed to team working as essential condition to achieve common corporate goals. At the same time, the technological dimension is perceived as less useful, probably due to the possibility of involving colleagues in solving difficulties with the employed devices as well as to the marginal role technology plays, if compared to the direct contact with other people. Such reflections are confirmed by the opposite positions occupied by the same Capabilities when referring to remote working; being able to deal with technology in order to maintain the same efficiency during the working activity at a distance is, in this case, fundamental. Agility and innovation are the other two Capabilities the participants have indicated as most useful working remotely, emphasizing how such new dimension requires both flexibility and adaptability, as well as the ability to implement innovative approaches and solutions, abandoning the comfort-zone and opening up to new ways. The imposed distance also impacts the human relationships, as this estranging working method forces to put into practice a more autonomous and independent management of the activities.

Without making distinctions concerning the type workplace, it can be observed how the rankings reflect the situation of the working *at a distance* scenario, stressing how the crucial role played by new technologies cannot be ignored:

**Table:** 

*Deal with technology*	6.22
*Agility*	6.21
*Engagement*	6.17
*Innovation*	6.09
*Collaboration*	6.03
*Interdisciplinarity*	6.03
*Deal with humans*	5.89

Furthermore, observing the evaluations provided by the participants belonging to different generations offers interesting insights into how the Capabilities are perceived:

**Table:** 

Z-Y Generations		X Generation – Baby Boomers	
*Deal with technology*	6.20	*Agility*	6.30
*Agility*	6.15	*Engagement*	6.27
*Engagement*	6.13	*Deal with technology*	6.26
*Innovation*	6.11	*Interdisciplinarity*	6.15
*Collaboration*	6.04	*Innovation*	6.08
*Interdisciplinarity*	6.00	*Collaboration*	6.04
*Deal with humans*	5.94	*Deal with humans*	5.80

It can be noticed how the younger generations assign higher evaluations to *deal with technology*, *innovation* and *collaboration*; on the contrary, the older generations place higher in the list *agility*, *engagement*, and *interdisciplinarity*. This is not surprising, as the Z-Y Generations are actually made up of individuals who have grown up in close contact with new technologies, an element that has always been part of their lives, and, at the same time, they are more used to working with others, in teams. Baby Boomers and the X Generation include on the other side people who are less accustomed to new techniques and innovative methods, who tend to maintain a more traditional working approach, regardless of generation is assigned a prominent position to both *Agility* and *Engagement*, proving that adaptability, flexibility, and passion are driving factors recognized as indispensable. It is interesting that *Interdisciplinarity* is ranked higher by the mature generations; this could be explained as a consequence of their less confidence with innovative technologies, requiring to be prepared on multiple fronts to be able to handle different issues in case of need.

Combining the answers of the different generations with the working locations, it can be observed how the priority of some Capabilities changes according to the situational context, reflecting, in any case, the abovementioned participants’ preferences and needs.

One of the open questions included in the survey provided additional relevant findings about the seven Capabilities in the context of the remote working experience, as it asked the participants to choose one of them and describe its effective application, mentioning a concrete example. The fact that most of the 500 workers talked about *Deal with Technology* confirms the abovementioned results, acknowledging that the technological dimension is essential and unavoidable working at a distance; video conferences, printing, scanning, using a personal computer, and the email are some things that, today, cannot be avoided to carry out every kind of working activity. Similarly, the examples provided concerning *Agility* support the already illustrated findings, as the participants highlighted the importance of being flexible and open-minded to solve complex problems and to adapt in order to meet new challenging requests. It cannot be denied that the surrounding environment has a deep impact on the working activity, which, if performed remotely, may undergo negative effects as the loss of both motivation and concentration; this has emerged from the words of those who chose an *Engagement* episode. *Cooperation* is then a Capability with a strong emotional component, recognized as fundamental from both the professional and personal perspectives; mutual support is pursued to overcome the physical distance and to obtain common positive results. Feeling part of a team, keeping the human aspect alive, is another recurring need expressed by the workers, who talked about the *Deal with Human* Capability, describing the difficulties generated by the imposition of a filter between individuals. A transformed surrounding scenario requiring new approaches and solutions, no more accepting the traditional ones, was then described by the participants who decided to share the implementation of *Innovation*. Just a minority talked about *Interdisciplinarity*, emphasizing the need to face complex situations in an autonomous way, being able to deal with different competence areas just relying on your own competences and experiences.

● 26.9% *Deal with Technology*: “*Indispensable to work remotely; indispensable to keep in touch with the colleagues; it allows to be in more place at the same time*.”
● 17.7% *Agility*: “*Ability to adapt to new requests, to be flexible, to define priorities; being able to balance your working and private life.*”
● 16.8% *Engagement*: “*Passion for your work stimulates motivation; to add something of your own is essential, aiming to obtain good results*.”
● 15.5% *Collaboration*: “*Essential for the creation of synergies, coordination, technical support; supporting and being supported, being patient.*”
● 12% *Deal with humans*: “*Good communication is crucial; maintaining positivity and humanity, being emphatic, being able to reassure; feeling part of a group, motivated.*”
● 9.5% *Innovation*: “*A new approach to face daily problems, reinventing yourself, abandoning the traditional ways.*”
● 1.6% *Interdisciplinarity*: “*Because of the distance*, *it is essential to be autonomous, being able to move from a topic to another without difficulties.*”

The survey also allowed the 500 participants to indicate other Capabilities they perceived as useful during their remote working experience; just 16% of them answered this question, referring to additional competences they considered worth mentioning. The majority talked about autonomy and independence (28.6%) in order to manage the activities effectively and to organize the tasks properly. A group of participants focused then on time management (17.4%) to be considered not just from the professional point of view but also in terms of intelligent planning of the private dimension. Communication skills (12.2%) have also been mentioned by some workers, as the distance imposed makes it more complicated to listen and trust the others in an appropriate way, as well as to focus on the other’s needs. Smaller groups included additional dimensions related to the emotional and personal spheres, talking about self-control, stress management, and patience (7%); empathy (2.6%); self-learning (0.8%); sharing of information, supporting others and being generous (0.8%).

**Table:** 

TARGET									

	All			Z-Y Generations			X Generation – Baby Boomers		
	Gen.	On Site	Dist.	Gen.	On Site	Dist.	Gen.	On Site	Dist.
*Deal with humans*		2°			1°				
*Deal with technology*	1°		1°	1°		1°	3°		1°
*Agility*	2°	3°	2°	2°	3°	3°	1°	2°	2°
*Engagement*	3°			3°			2°	1°	3°
*Collaboration*		1°			2°			3°	
*Interdisciplinarity*									
*Innovation*			3°			2°			

Finally, a specific section of the questionnaire aimed at investigating the perception of the remote working experience from a more general perspective, asking the involved workers to express an evaluation about it with regard to the professional and private spheres.

**FIGURE 7:**
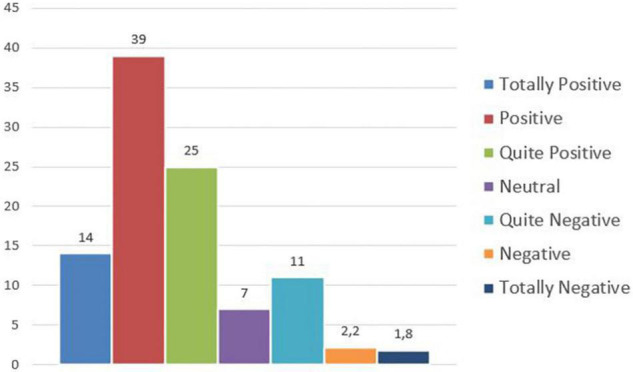
Remote working experience perception – professional point of view.

The evaluations provided from the *professional* point of view can be classified as follows in Figure 7.

**FIGURE 8:**
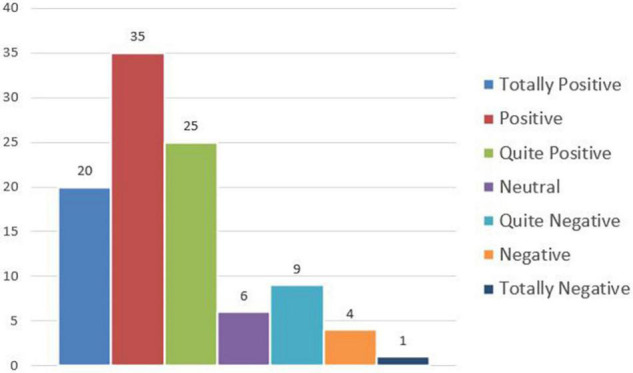
Remote working experience perception – personal point of view.

The evaluations provided from the *personal* point of view can be instead classified as follows in Figure 8.

On the whole, 78% of the sample has appreciated the remote working experience from the professional point of view and 80% from the personal point of view; the same situations have been perceived negatively from about 15% of the participants, and about 6% is neutral. The main advantages of working at a distance include a reduction of the wasted time spent moving and more satisfaction for their condition, elements that can both originate improved productivity and performance; referring to the private dimension, some benefits correspond to self-management of time and opportunity to choose the working place, as well as more free time and the possibility to stay close to family and friends; the general aspect that is more appreciated is the increased flexibility allowed, also affecting the motivational factor positively.

Reconnecting to the future scenario, the survey also proposed a question with the purpose of analyzing the propensity to prefer working at a distance, if being able to choose for the future, selecting one of the following six options:

**Table:** 

Yes, every day	10%
Yes, more than 2 days a week	20.2%
Yes, 2 days a week	29.3%
Yes, 1 day a week	15.5%
Yes, just if necessary	17%
No	7%

Just a minority would not continue working at a distance in the future, while 93% of the sample would, the preferred solution would be around 2 or more days a week, therefore quite frequently. Such data provide significant evidence about the average population, which seems ready for this deep transformation in the working routine, entailing a strong impact on the traditional corporate weekly schedule. The COVID-19 emergency has probably let this aspect emerge, bringing to light also in Italy the willingness to adopt a new working mode that is already widespread in other countries.

Some interesting observations emerge when analyzing the responses given by different generations:

**Table:** 

Z-Y Generations		X Generation – Baby Boomers	
Yes, 2 days a week	34%	Yes, just if necessary	22%
Yes, more than 2 days a week	19%	Yes, more than 2 days a week	21%
Yes, 1 day a week	18%	Yes, 2 days a week	19%
Yes, just if necessary	15%	Yes, every day	17%
Yes, every day	7%	Yes, 1 day a week	11%
No	7%	No	10%

With regard to younger generations X and Z, the majority chose “2 days a week”; conversely, generations X and Baby Boomers “just if necessary.” Although, in both cases, working at a distance is generally fully appreciated, we can deduce that the younger generations are more comfortable with this innovative mode of working.

The findings presented allow to conclude that the pandemic has inevitably made the need to develop and implement these Capabilities more evident, accelerating their adoption into everyday work in a much shorter time than we might have expected. The importance of taking advantage of the opportunities generated by new technologies has emerged, besides the acknowledgment of flexibility and open-mindedness as crucial factors to face an ever-changing context. The usefulness of this set of Capabilities thus seems to be confirmed by the results of the survey, which highlights the crucial role they are playing since the outbreak of the pandemic, and which they seem to suggest will remain even once the pandemic is over. Regardless to the significant role increasingly played by technological innovation, the results of this study have also highlighted one remarkable element: how the human factor remains fundamental and how attention to people’s needs must not be put aside, even when work is done remotely, requiring this dimension to be integrated.

## Conclusion

The study conducted with the purpose of answering the two research questions has provided an interesting insight into the renewed working experience generated by the COVID-19 pandemic outbreak. Both the external desk-based research and the survey allow to draw some conclusions that can be considered consistent with the research hypothesis. Concerning the first research question, investigating the skills emerged as necessary elements of the working experience during the pandemic, a new concept of Capability has emerged, a combination of knowledge, ability, and experience useful to people in every aspect of daily life in order to participate consciously in the surrounding environment that they are living, as well as to create a new future dimension with both intention and full awareness. In particular, a set of seven Capabilities whose development has been accelerated by the pandemic has been identified in the Comau context, as suitable expression of the renewed approach to the working activity, meeting the renewed everyday requirements: people and their needs have to be put at the center (*Deal with Humans*); the confidence with new technologies is unavoidable (*Deal with Technology*); it is crucial to be open-minded and flexible (*Agility*); a real passion enhances every outcome (*Engagement*); it is essential to constantly cooperate with the others (*Collaboration*); different kinds of knowledge have to be combined to achieve relevant results (*Interdisciplinarity*); new possibilities and solutions have to be continuously pursued (*Innovation*). Obviously, the capabilities are not intended to be predictive of the future scenario, which will inevitably be additionally influenced by technological, economic, and social factors; this study merely identifies the key elements of the new work culture that emerged at the time of the research.

Regarding the usefulness of the seven Capabilities as key elements of a new culture of work during the pandemic and, as stated in the second research question, also for a post-pandemic future, it has been undertaken through the analysis of the Comau case. The vast majority of the workers participating in the conducted survey, involving 500 people who started working at a distance during the 2020 lockdown period, have defined the seven Capabilities as useful or very useful during the global crisis they had to face; 90% of them think that such Capabilities will be also useful in the future. The Capabilities will be in any case just one of the ingredients that will characterize the new work culture; a crucial role will be inevitably played by business organization, process aspects, and hard skills.

The seven Capabilities have been all described in a positive way, but some of them have been better taken into account; the familiarity with the technological dimension (*Deal with Technology*) is necessarily the one recognized as indispensable for each kind of activity, in particular when working at a distance. *Agility* and *Engagement* follow, as, in the current constantly changing scenario, is essential to be flexible and rapid to react proactively and effectively, always moved by passion and interest. *Innovation*, *Collaboration*, and *Interdisciplinarity* confirm the perception of a context where the urgency is to seize the opportunities offered by the digital revolution without losing the human dimension (*Deal with Humans*) in terms of relationships, humanity, and exchange between individuals.

The human beings must, therefore, be put at the center in order to guide the technological progress and manage its innovation, both at organizational and social levels; workers have to be responsible for their choices and to implement new instruments; at the same time, as individuals, they have to be aware of their roles in such a scenario. The main purpose corresponds to the search for balance between technology and centrality of the human factor, allowing the best co-existence of the two spheres, in a context where workers can use digital devices to improve their performances as functional to the needs of people, without perceiving technology as an external and overwhelming element. Aware of the limitations of the research presented in this article, it is planned to carry out a future experimental study that will further investigate the raised issues.

The conclusions illustrated in this final section of the article offer then some concrete indications and coordinates for the subjects involved in the training and education sectors, who face the deep transformation of the surrounding context without full awareness of its impacts. Institutions, stakeholders, and who provide/participate in training initiatives may, therefore, take advantage of this research, considering the evident need of raising consciousness about the new culture of work and the required Capabilities in order to support people performing their working activity effectively and becoming successful protagonists of both the current and future scenarios.

## Data Availability Statement

The raw data supporting the conclusions of this article will be made available by the authors, without undue reservation.

## Ethics Statement

The studies involving human participants were reviewed and approved by Internal Control Committee of COMAU SpA. The patients/participants provided their written informed consent to participate in this study.

## Author Contributions

All authors listed have made a substantial, direct, and intellectual contribution to the work, and approved it for publication.

## Conflict of Interest

EF was employed by the company Comau S.p.A. The remaining authors declare that the research was conducted in the absence of any commercial or financial relationships that could be construed as a potential conflict of interest.

## Publisher’s Note

All claims expressed in this article are solely those of the authors and do not necessarily represent those of their affiliated organizations, or those of the publisher, the editors and the reviewers. Any product that may be evaluated in this article, or claim that may be made by its manufacturer, is not guaranteed or endorsed by the publisher.

## Footnotes

1
https://www.simon-kucher.com/it/about/media-center/coronavirus-11-world-economy-thriving-alarming-58-threatened

2
https://www.bancaditalia.it/pubblicazioni/note-covid-19/2020/Nota-COVID-Statistiche-2020.10.22.pdf

3
https://www.eurofound.europa.eu/publications/report/2017/working-anytime-anywhere-the-effects-on-the-world-of-work
